# Clinical and Prognostic Significance of HIF-1α, PTEN, CD44v6, and Survivin for Gastric Cancer: A Meta-Analysis

**DOI:** 10.1371/journal.pone.0091842

**Published:** 2014-03-19

**Authors:** Jing Chen, Tao Li, Qilun Liu, Haiyan Jiao, Wenjun Yang, Xiaoxia Liu, Zhenghao Huo

**Affiliations:** 1 Department of Medical Genetic and Cell Biology, Ningxia Medical University, Yinchuan, China; 2 Key Laboratory of Fertility Preservation and Maintenance (Ningxia Medical University), Ministry of Education, Yinchuan, China; 3 Department of Oncology, General Hospital of the Ningxia Medical University, Yinchuan, China; Sapporo Medical University, Japan

## Abstract

**Purpose:**

This study was to quantitatively summarize published data for evaluating the clinical and prognostic significance of four proteins involved in hypoxia-inducible factor-1 (HIF-1α) regulation of the metastasis cascade.

**Methods:**

Searches were performed using the MEDLINE, EMBASE, Cochrane Library, and Chinese Biomedicine databases without any language restrictions. Studies were pooled and either the summary risk ratio (RR) or odds ratio (OR) was calculated. Potential sources of heterogeneity were sought out *via* subgroup and sensitivity analyses, and publication bias was also performed.

**Results:**

Seventeen studies evaluated HIF-1α, 20 studies evaluated phosphatase and tensin homolog (PTEN), 20 studies evaluated Survivin, and 16 studies evaluated CD44v6. Our results showed that increased HIF-1α expression was linked to a poor 5-year overall survival (RR = 1.508; 95% confidence interval (CI) 1.318–1.725; P<0.001). Decreased survival was heavily influenced by advanced tumor invasion (OR = 3.050; 95% CI 2.067–4.501; P<0.001), lymph node metastasis (1415 patients; OR = 3.486, 95% CI 2.737–4.440; P<0.001), distant metastasis (OR = 6.635; 95% CI 1.855–23.738; P = 0.004), vascular invasion (OR = 2.368; 95% CI 1.725–3.252; P<0.001), dedifferentiation (OR = 2.112; 95% CI 1.410–3.163; P<0.001), tumor size (OR = 1.921; 95% CI 1.395–2.647; P<0.001), and a higher TNM stage (OR =  2.762; 95% CI 1.941–3.942; P<0.001). Similarly, aberrant expression of PTEN, CD44v6, and Survivin were also observed in tumors that correlated with poor OS. The higher ORs of death at 5 years were 1.637 (95% CI = 1.452–1.845; P<0.001), 1.901 (95% CI = 1.432–2.525; P<0.001), and 1.627 (95% CI = 1.384–1.913; P<0.001), respectively, with an OR>2 for the main stratified meta-analyses of clinical factors.

**Conclusions:**

Our findings indicate that HIF-1α/PTEN/CD44v6/Survivin, as measured by immunohistochemistry, can be used to predict the prognosis and potential for invasion and metastasis in Asian patients with gastric cancer. The development of strategies against this subset of proteins could lead to new therapeutic approaches.

## Introduction

Gastric cancer is one of the most aggressive tumors and tends to be associated with peritoneal dissemination, lymph node metastasis, and hematogenous metastasis. Although recent advances in its diagnosis and treatment have offered increased long-term survival for patients diagnosed at early stages of gastric cancer, the prognosis of advanced cancer remains dismal, with a 5-year survival rate of only 10–15% [1], [2]. A majority of patients with advanced disease die due to complications induced by metastasis but not the primary tumor [3]. Recently, a series of rate-limiting steps have been proposed for tumor cells to become metastatic [4]. The multi-step processes consist of loss of cellular adhesion, local invasion, motility, angiogenesis, intravasation, circulation, extravasation, homing and the premetastatic niche, and organotropic colonization in specific organs [5]. Therefore, identifying novel markers in the key steps of metastasis will help to predict recurrence and survival for patients in the early stages of gastric cancer.

Hypoxia has been reported to contribute directly to many critical aspects of cancer biology, including angiogenesis, epithelial-mesenchymal transition, invasion, metastasis, stem cell maintenance, energy metabolism, autocrine growth factor signaling, and refractory to targeted therapies [6], [7]. The best characterized hypoxia response pathway is mainly mediated through a transcription factor called hypoxia-inducible factor-1 (HIF-1α) [8]. Currently, the number of target genes, which are controlled by HIF-1α, is greater than 1000 and can be divided into the following five categories: transcription factors and histone modifiers; matrix degradation enzymes; receptor, receptor-associated kinases, and transporters; microRNA targets; and cell-adhesion molecules and membrane proteins [9], [10]. In addition, routine phase 1 and phase 4 clinical trials that target HIF-1α function or expression have been completed, including a pilot trial of oral Topotecan for the treatment of refractory advanced solid neoplasms expressing HIF-1α and the effects of Dutasteride on HIF-1α and vascular endothelial growth factor (VEGF) in the prostate (Clinical Trial: NCT00117013, NCT00880672; http://clinicaltrials.gov/). The positive results from these clinical trials have further reinforced the interest in drug development targeting HIF-1α signaling.

Despite the clinical development of anti-HIF-1α therapies, the prognostic and clinical value of HIF-1α overexpression in gastric cancer cells remains unclear. It is essential to explore whether tumors in which HIF-1α is overexpressed are associated with reduced survival. As the incidence and mortality rate of gastric cancer are extremely higher in Eastern Asian especially China, Japan and Korea, we present a meta-analysis evaluating the prognostic impact of one subset of proteins in HIF-1α signaling in gastric cancer patients in subgroup of different continents. We hope that our meta-analyses will provide a framework for hypoxia regulation of the metastasis cascade and further uncover the role of hypoxia/HIF-1α-regulated key target genes on the prognosis based on various steps of metastasis. Most importantly, the analyses of gene expression profiles on prognosis may lead to the development of clinical methods that can be used to predict the outcome of individual patients in a clinical setting.

## Methods

### Search strategy and selection criteria

The meta-analysis was performed by means of preferred reporting items for meta-analyses statement [11], [12]. The PUBMED, EMBASE, Cochrane Library, and Chinese National Knowledge Infrastructure (CNKI) databases were searched (up until June 2013) without language restrictions. Various combinations of the following terms were used to screen for potentially related studies: “prognosis” and “survival” and “gastric” or “stomach” as well as “cancer” or “carcinoma” or “tumor”.

Studies were included in the meta-analysis if they met the following criteria: (1) diagnosed gastric cancer and normal gastric epithelial mucosa in humans; (2) evaluated proteins by using immunohistochemistry (IHC) methods; (3) used Asian cohorts from medical centers, and (4) had a follow up time exceeding 5 years. The study selection was based on the association of proteins related to HIF-1α-mediated tumor metastasis signaling and prognosis. References of retrieved articles were cross-searched to identify any studies missed by the computerized literature search. Authors of eligible studies were contacted for additional data relevant to the meta-analysis. However, not all authors responded.

### Data extraction and methodological assessment

Data retrieved from all full publications included author, year of publication, country, antibody used for evaluation, and cut-off for diagnosis based on abnormal protein expression. In addition, data was collected on the number of readers, blinded readings, number of controls and cases, depth of invasion, lymph node status, distant metastasis, TNM stage, vascular invasion, histo-differentiation, tumor size, sex, and age of gastric cancer patients. Overall survival is defined as the time elapsed from surgery to death of patients with gastric cancer. In all cases, the data of interest for 5-year survival rates were extracted from Kaplan-Meier curves.

We tried to carefully avoid the duplication of data by examining each publication, the names of all authors, and the different medical centers involved. When an individual author published several articles obtained from the same or overlapping population, only the newest or most complete article was included in the analysis; otherwise independent data were analyzed. All data were extracted independently by three investigators (Chen J, Li T, and Liu XX), and any disparities were resolved by discussion.

### Statistical analysis

In the present study, we analyzed three categories of stratified models. The first stratified multivariate model was performed to confirm whether each protein was abnormally expressed in gastric cancer compared to the normal gastric mucosa. The second outcome of the meta-analysis was to measure the impact of aberrant protein expression on 5-year overall survival. The third model was used to examine the prognostic value of protein expression that was corrected by clinical variables, including sex, age, histo-differentiation, tumor size, depth of invasion, vascular invasion, lymph node status, distant metastasis, and TNM stage.

Based on clinical characteristics, the following variables were compared: T_1_ and T_2_ vs. T_3_ and T_4_; Stage I and Stage II, vs. Stage III and Stage IV; well and moderate differentiation vs. poor and undifferentiation; tumors larger than 5 cm in size vs. tumors less than 5 cm; and patients older than 60 years vs. patients younger than 60 years.

Data were combined to perform meta-analysis using STATA version 9.0, and a two-tailed p-value of less than 0.05 was considered to be statistically significant. Estimates of risk ratios (RRs) or odds ratios (ORs) were weighted and pooled using different models based on their extent of heterogeneity. The heterogeneity across studies was quantified by using the I^2^ statistic, which is generally considered significant for values ≥50%. In the case of heterogeneity, meta-analysis was performed using the random effects model after exploring the causes of heterogeneity. Otherwise, the fixed-effects model was applied. In addition, one-way sensitivity analysis was conducted to assess the stability of the results, namely, a single study in the meta-analysis was deleted one at a time to check the influence of the individual data set on the pooled RR (or OR) [13], [14]. Both Begg's funnel plot and Egger's test were used to determine any publication bias for each of the pooled study groups.

## Results

### Study selection and characteristics

**Figure 1:**
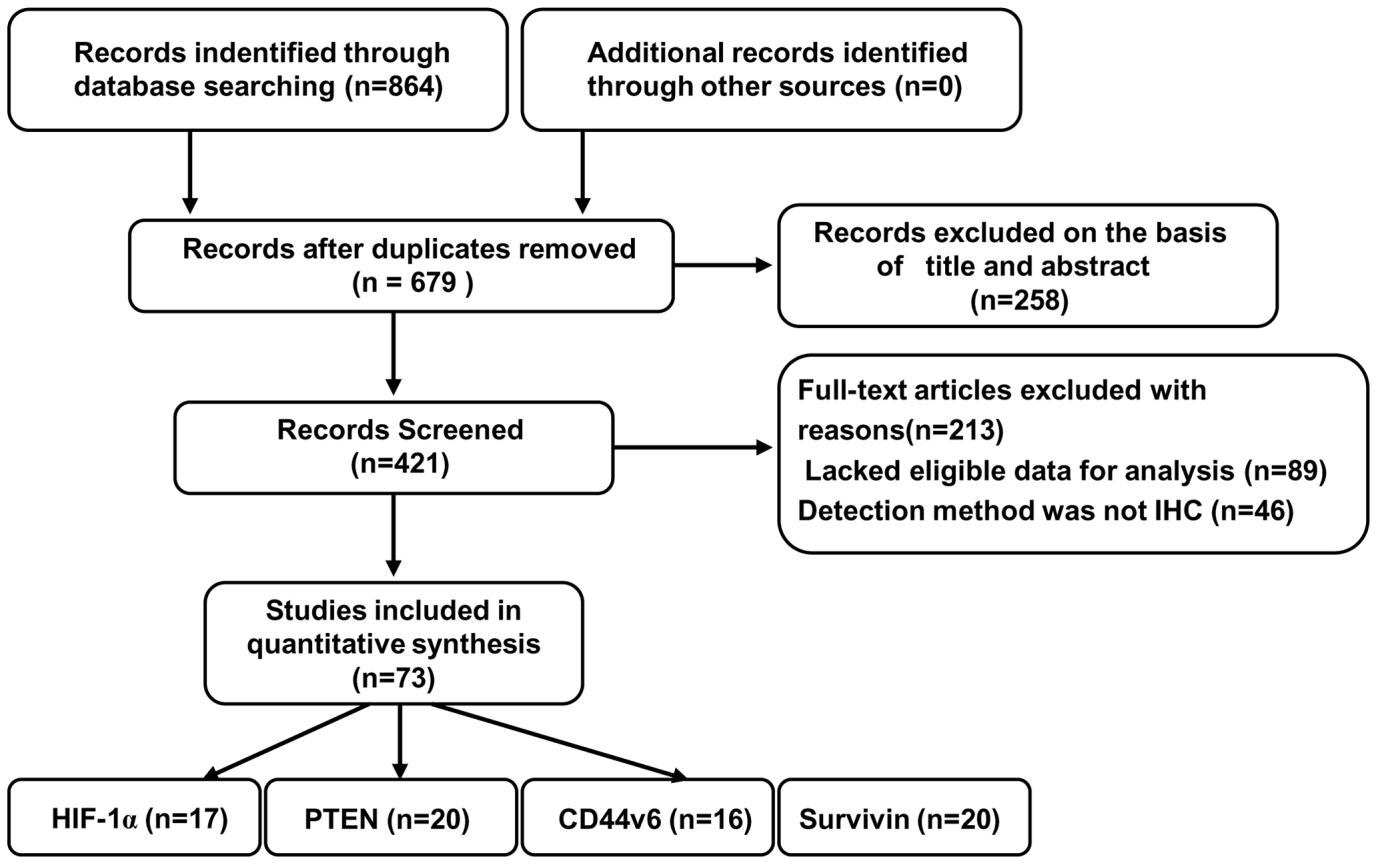
Flow chart of the meta-analysis.

After screening a collection of publications on proteins associated with hypoxia regulation of the metastasis cascade and prognosis, we identified 73 studies that used IHC techniques to assess the expression of four proteins on our prior criteria in the search strategy and study selection. Of these, 17 studies evaluated HIF-1α (from Liu et al., 2004 to Hoon Hur, et al., 2013), 20 studies evaluated phosphatase and tensin homolog (PTEN) (from Li et al., 2003 to Hye Seung Lee et al., 2003), 20 studies evaluated Survivin (from Yu et al., 2002 to Min A Kim et al., 2011), and 16 studies evaluated CD44v6 (from Xin et al., 2001 to Dae-Woon Eom et al., 2011) (Fig. 1, Table 1). The median age of patients was 57.7 years old. The main characteristics of included studies are shown in Table 1 and Table S1, and the publications used to perform meta-analyses are listed in the References S1.

**Table 1:** Main characteristics of the 73 studies included in the final meta-analyses.

First author		Language	Race	Study	Number			Cutoff	Protein	Antibody					
of issue	Pub			From	of patients	Media age	Antibody	for protein	abnormal	used for	Blinded	Reader(s)	RR	Survival	Results
(reference)	(Year)			Pubmed	(M/F)	(years)	dilution	positivity (%)	Exp (%)	evaluation	reading	(n)	Estimate	analysis	
Liu, et al	2004	Chinese	China	No	-	-	1:100	-	63.3%	Rb, Boster Biotech	-	-	-	-	-
Chen, et al	2005	English	China	Yes	34/28	-	1:50	>10%	-	H-206, Santa Cruz, CA	-	-	-	-	-
Han, et al	2006	Chinese	China	Yes	58/38	61.5	1:50	>10%	80.2%	MS-1164-P0, NeoMarkers	-	-	Reported in text	OS	Positive
Ru, et al	2007	Chinese	China	Yes	86/32	57.6	-	>10%	-	-	Yes	2	Reported in text	OS	Positive
Wu, et al	2009	Chinese	China	Yes	34/18	58	-	>10%	53.9%	Rb, Boshide Biotech	-	-	-	-	-
Qiu, et al	2011	English	China	Yes	127/61	57	1:50	-	58.5%	MS-1164-P0, NeoMarkers	Yes	2	Reported in text	OS	Positive
Wang, et al	2010	English	China	Yes	59/21	-	1:200	>10%	-	MS, Chemicon	-	-	-	-	-
Jia, et al	2013	English	China	Yes	117/56	62	1:50	>10%	-	MS, Santa Cruz, CA	Yes	2	-	-	-
Lu, et al	2013	English	China	Yes	43/25	49.9	1:100	>6%	-	MS, MAB1935 R and D	Yes	2	Reported in text	OS	Negative
KEN MIZOKAMI, et al	2006	English	Japan	Yes	85/41	65.2	1:100	>10%	38.9%	NB 100-105, Novus Biologicals, CO	Yes	2	Reported in text	OS	Positive
Yasushi Sumiyashi, et al	2006	English	Japan	Yes	148/68	65.2	1:100	>10%	39.4%	NB 100-105, Novus Biologicals, CO	Yes	2	Reported in text	OS	Positive
NaoMI URANO, et al	2006	English	Japan	Yes	-	-	1:50	>10%	-	H1α 67, Novus Biologicals, CO	-	-	Reported in text	OS	Negative
Yanislav Kolev, et al	2008	English	Japan	Yes	110/42	59.5	1:500	>10%	62.5%	MS, MAB 5382	Yes	2	Reported in text	OS	Negative
JUN Nakamura, et al	2009	English	Japan	Yes	43/20	66.9	1:200	>10%	57.1%	NB 100–105, Novus Biologicals, CO	-	-	Reported in text	OS	Positive
Taro Isobel, et al	2012	English	Japan	Yes	91/37	67.3	1:50	>5%	-	H-206, Santa Cruz, CA	-	-	Reported in text	OS	Positive
SunYong Oh, et al	2008	English	Korea	Yes	67/47	59	1:50	>1%	-	H1α 67, Novus Biologicals, CO	-	-	Reported in text	OS	Positive
Hoon hur, et al	2013	English	Korea	Yes	96/56	-	1:50	>5%	-	Thermo Fisher Scientific, CA	Yes	2	Reported in text	OS	Negative
Li, et al	2003	Chinese	China	No	70/30	56	-	>10%	-	Rb, Boster Biotech	-	-	Reported in text	OS	Positive
Yang, et al	2003	English	China	Yes	-	-	-	>5%	52.2%	Rb, Maixim Biotech	-	-	-	-	-
Zheng, et al	2003	English	China	Yes	83/30	57.1	-	>5%	45.1%	Antibody Dignostica	Yes	2	-	-	-
Li, et al	2004	Chinese	China	Yes	-	-	-	>5%	70.2%	MS, Maixim Biotech	-	-	-	-	-
Deng, et al	2006	English	China	Yes	99/41	58	-	-	-	Clone 17A, NeoMarkers	-	-	-	-	-
Li, et al	2007	Chinese	China	Yes	112/56	63	-	>5%	53.0%	MS, Maixim Biotech	-	-	Reported in text	OS	Positive
Wang, et al	2008	English	China	No	73/39	49.4	-	-	62.5%	Shanghai Changdao Biotech	Yes	2	-	-	-
Guo, et al	2008	English	China	Yes	41/12	65.6	-	>10%	34.0%	-	-	-	-	-	-
Wu, et al	2011	English	China	Yes	-	-	-	-	-	Zymed Laboratories Inc	Yes	2	Reported in text	OS	Negative
Liang, et al	2012	English	China	Yes	55/26	55.4	1:100	>10%	-	Clone 28H6, Invitrogen Co	Yes	2	Reported in text	OS	Positive
Yang, et al	2012	English	China	Yes	23/20	-	1:150	>5%	60.0%	ab31392, Abcam	Yes	2	-	-	-
Li, et al	2012	English	China	Yes	15/18	51.6	-	>10%	54.6%	MS, Maixim Biotech	Yes	3	-	-	-
Bai, et al	2013	English	China	Yes	170/58	60.7	-	>10%	-	-	Yes	2	Reported in text	OS	Positive
Zhu, et al	2013	English	China	Yes	112/47	65	1:50	>5%	61.6%	Ms, Santa Cruz, CA	Yes	3	-	-	-
Zheng, et al	2007	English	Japan	Yes	177/72	66.6	1:200	>5%	27.7%	NovoCastro, Newcastle, UK	Yes	3	Reported in text	OS	Positive
Rumi Hino, et al	2009	English	Japan	Yes	65/29	-	1:100	>10%	-	MS, Santa Cruz, CA	-	-	-	-	-
Young-Hwa Kang, et al	2002	English	Korea	Yes	212/98	54.4	1:200	>10%	20.0%	MS, AG Science,	-	-	Reported in text	OS	Positive
Seock Ah Im, et al	2003	English	Korea	Yes	52/42	59	1:100	>10%	-	MS, Neomarker, Fremont	-	-	Reported in text	OS	Positive
Geun Soo Park, et al	2005	Korean	Korea	Yes	57/33	-	1:200	>10%	-	NeoMarkers, Union, CA	-		-	-	-
Hye Seung Lee, et al	2003	English	Korea	Yes	-	54.8	1:50	>10%	-	San Diego, CA	-	-	Reported in text	OS	Positive
Yu, et al	2002	English	China	Yes	33/17	62.2	1:200	>5%	58.3%	Novus Biologicals, Littleton, CA	Yes	2	-	-	-
Zhu, et al	2003	English	China	Yes	46/10	59.8	-	-	48.2%	-	-	-	-	-	-
Sun, et al	2003	Chinese	China	No	96/44	55.5	-	>5%	-	MS, NeoMarkers	-	-	Reported in text	OS	Positive
Yao, et al	2004	Chinese	China	Yes	72/48	64.8	1:50	>5%	-	MS, NeoMarkers	-	-	-	-	-
Li, et al	2004	English	China	Yes	56/24	60	-	>5%	76.3%	MS, NeoMarkers	-	-	-	-	-
Lu, et al	2004	Chinese	China	Yes	20/12	56	-	>10%	62.5%	MS, Santa Cruz, CA	-	-	-	-	-
Deng, et al	2006	English	China	Yes	99/41	58	-	-	-	RAB-0536, NeoMarkers	-	-	-	-	-
Sun, et al	2006	Chinese	China	Yes	73/24	55	1:100	>10%	60.8%	MS, Boster Biotech	-	-	Reported in text	OS	Positive
Cheng, et al	2007	English	China	Yes	29/24	-	-	-	100%	MS, Boster Biotech	-	-	-	-	-
Han, et al	2007	English	China	Yes	-	-	1:80	-	100%	Rb, Santa Cruz, CA	Yes	2	-	-	-
Deng, et al	2010	English	China	Yes	37/16	55	1:50	>5%	73.6%	Rb, Jingmei Biotechnology	Yes	2	Reported in text	OS	Positive
Li, et al	2010	English	China	Yes	38/27	56.2	1:100	>5%	-	Rb, Santa Cruz, CA	Yes	2	-	-	-
Meng, et al	2012	English	China	Yes	67/23	-	-	>5%	-	Rb, Santa Cruz, CA	-	-	Reported in text	OS	Positive
Deng, et al	2012	English	China	Yes	60/23	58	-	-	-	RAB-0536, NeoMarkers	-	-	-	-	-
Cai De Lu, et al	1998	English	Japan	Yes	124/50	59.7	-	>5%	34.5%	-	-	-	-	-	-
Rumi Hino, et al	2008	English	Japan	Yes	84/33	-	1:500	>10%	-	MS, LSAB2 Kit, Dako	-	-	-	-	-
Hirokazu Okayama, et al	2009	English	Japan	Yes	91/44	63.4	1:1000	-	-	Rb, Novus Biologicals, Littleton, CO	Yes	2	-	-	-
Gi-Hoon Lee, et al	2006	English	Korea	Yes	74/32	58.9	1:500	>10%	50.0%	R&D Systems Inc	Yes	2	Reported in text	OS	Positive
Kyo Yong Song, et al	2009	English	Korea	Yes	102/55	57.8	1:1000		40.1%	Rb, Novus Biologicals, Littleton, CO	Yes	2	Reported in text	OS	Positive
Min A Kim, et al	2011	English	Korea	Yes	774/388	-	1:400	>10%	-	R&D Systems Inc	-	-	-	-	-
Xin, et al	2001	English	China	Yes	-	-	1:200	>5%	40.7%	R&D Systems Inc	Yes	2	-	-	-
Li, et al	2003	Chinese	China	Yes	81/29	52.5	-	>5%	-	MS, Santa Cruz, CA	-	-	Reported in text	OS	Positive
Chen, et al	2004	English	China	Yes	26/17	58.5	-	>10%	-	MS, Maixin Biotech	-	-	-	-	-
Zhao, et al	2005	Chinese	China	Yes	27/13	52	-	>5%	-	MS, Jinshan Biotech, CA	-	-	-	-	-
Liu, et al	2005	English	China	Yes	26/14	-	-	-	62.5%	-	-	-	-	-	-
Lou, et al	2005	English	China	Yes	-	41	-	>5%	79.0%	Boster Biotech, CA	-	-	-	-	-
Han, et al	2007	English	China	Yes	-	-	1:100	-	83.9%	MS, -	Yes	2	-	-	-
Zhou, et al	2007	Chinese	China	Yes	72/31	56	1:100	>10%	60.2%	Zhongshan, Biotech, CA	-	-	Reported in text	OS	Negative
Liang, et al	2012	English	China	Yes	38/21	61.8	-	>5%	64.4%	MS, -	-	-	-	-	-
Ru, et al	2012	English	China	Yes	36/9	62.6	-	-	-	MS, Maixin Biotech	Yes	2	-	-	-
Kazushi Kurozumi, et al	1998	English	Japan	Yes	68/30	61	-	-	69.4%	-	-	-	-	-	-
Hiroaki Saito, et al	1998	English	Japan	Yes	75/42	62.6	-	>5%	-	-	-	-	Reported in text	OS	Positive
Akio Yamaguchi, et al	2002	English	Japan	Yes	-	-	-	-	-	-	Yes	2	Reported in text	OS	Positive
Hirokazu Okayama, et al	2009	English	Japan	Yes	91/44	63.4	1:100	>5%	62.2%	R&D Systems Inc	Yes	2	-	-	-
MeeJoo, et al	2003	English	Korea	Yes	59/40	57.6	-	>10%	68.7%	Clone, DF1485, Biogenex	-	-	-	-	-
Dae-Woon Eom, et al	2011	English	Korea	Yes	54/18	62	-	>10%	-	-	-	-	Reported in text	OS	Positive

Pub, publication; Exp, expression; RR, risk ratio; OS, overall survival; Positive, inverse relationship between specific protein expression and survival; Negative, no relationship. ‘Reader’ are readers of the histologic slides, ‘blinded reading’ means that readers of the slides without knowledge of the clinical outcome, and ‘−’ corresponds to missing data.

### Evaluation and expression of four specific proteins

Various antibodies were used to assess HIF-1α/PTEN/CD44v6 /Survivin expression. Among the group determined as HIF-1α-overexpressed, five studies used antibody NB-100-105 (Novus Biologicals; CA) and three studies used antibody H-206 (Santa Cruz Biotechnologies; CA) (Table 1). The cut-off points for overexpression of HIF-1α selected in most studies was 10% in terms of antibody dilution ranging from 1∶50 to 1∶100 (Table 1). The median frequency for the subset of proteins expressed in gastric cancer was 54.1% (range, 38.9–80.2%) for HIF-1α, 61.2% (range, 40.7–3.9%) for CD44v6, 55.6% (range, 34.5–76.3%) for Survivin, and 43.3% (range, 20.0–70.2%) for PTEN. A description of the antibodies used in the included studies is shown in Table 1.

### Meta-analysis results

#### Correlation of HIF-1α expression between gastric cancer and normal gastric mucosa

Our analyses, combining 8 independent studies that included 923 patients and 898 controls, revealed that HIF-1α overexpression was frequently observed in patients with gastric cancer compared to the counterpart normal tissue. The OR was 272.194 (95% confidence interval (CI) 99.702–743.112, P<0.001), without any heterogeneity between studies (I^2^ = 0.00%, subgroup difference P = 0.920) (Table 2).

**Table 2:** Meta-analyses of HIF-1α/PTEN/CD44v6/Survivin expressions on gastric cancer.

Stratification of gastric cancer	HIF-1α											PTEN							
	Nation	NS	NP	Model	OR(RR) (95%CI)		P		I^ 2^	P _bias_	Nation	NS		NP	Model	OR(RR) (95%CI)	P	I^ 2^	P_bias_
Case-Control	China	4	707	Fixed	283.675 (68.842–1168.933)	0.000		0.00%		0.096	China		9	1660	Random	18.197 (10.201–32.462)	0.000	57.10%	
	Japan	4	1114	Fixed	263.152 (64.237–1078.016)	0.000		0.00%			Japan		1	382	-	3.538 (1.873–6.684)	0.000	.%	
	Korea	-	-	-	-	-		-			Korea		1	620	-	156.187 (9.614–2537.471)	0.000	.%	
	All	8	1821	Fixed	272.194 (99.702–743.112)	0.000		0.00%			All		11	2662	Random	16.930 (8.613–33.280)	0.000	75.00%	0.034
Overall 5-year survival	China	3	350	Fixed	1.486 (1.191–1.855)	0.000		0.00%		0.331	China		5	605	Random	1.532 (1.309–1.792)	0.000	59.20%	
	Japan	5	717	Fixed	1.554 (1.264–1.911)	0.000		21.20%			Japan		1	232	-	1.645 (1.275–2.123)	0.000	.%	
	Korea	2	266	Random	1.428 (1.072–1.902)	0.015		87.00%			Korea		3	714	Fixed	1.839 (1.424–2.376)	0.000	0.00%	
	All	10	1333	Fixed	1.508 (1.318–1.725)	0.000		35.30%			All		9	1551	Fixed	1.637 (1.452–1.845)	0.000	40.60%	0.006
The depth of invasion	China	4	452	Fixed	5.046 (2.867–8.880)	0.000		35.70%		0.017	China		9	956	Random	1.862 (1.036–3.346)	0.038	62.30%	
	Japan	4	622	Fixed	1.926 (1.384–2.680)	0.000		0.00%			Japan		1	249	-	5.085 (2.698–9.586)	0.000	.%	
	Korea	1	114	-	4.231 (1.482–12.079)	0.007		.%			Korea		2	394	Fixed	6.149 (3.135–12.062)	0.000	0.00%	
	All	9	1188	Random	3.050 (2.067–4.501)	0.000		53.80%			All		12	1599	Random	2.604(1.554–4.366)	0.000	69.00%	0.909
Lymph node status	China	6	683	Random	3.814 (2.703–5.382)	0.000		59.30%		0.060	China		13	1410	Random	2.499 (1.704–3.665)	0.000	52.60%	
	Japan	4	618	Fixed	3.195 (2.248–4.542)	0.000		0.00%			Japan		2	364	Random	3.010 (1.250–7.251)	0.014	60.20%	
	Korea	1	114	-	3.276 (0.888–12.084)	0.075		.%			Korea		3	484	Random	2.136 (0.988–4.617)	0.054	62.90%	
	All	11	1415	Fixed	3.486 (2.737–4.440)	0.000		31.40%			All		18	2258	Random	2.484 (1.836–3.360)	0.000	51.50%	0.583
Distant metastasis	China	4	341	Random	6.617 (1.273–34.391)	0.025		79.90%		0.711	China		5	650	Fixed	2.947 (1.605–5.411)	0.01	0.00%	
	Japan	1	128	-	6.563 (1.457–29.551)	0.014		.%			Japan		1	249	-	8.345 (2.558–27.218)	0.000	.%	
	Korea	-	-	-	-	-		-			Korea		3	494	Fixed	1.485 (0.803–2.746)	0.207	47.40%	
	All	5	469	Random	6.635 (1.855–23.738)	0.004		73.30%			All		9	1393	Fixed	2.528 (1.703–3.751)	0.000	35.30%	0.818
TNM stage	China	6	665	Fixed	3.602 (2.439–5.320)	0.000		21.30%		0.068	China		8	904	Fixed	2.019 (1.247–3.267)	0.004	44.70%	
	Japan	3	495	Fixed	1.602 (1.097–2.338)	0.015		0.00%			Japan		1	115	-	3.339 (1.131–9.858)	0.029	.%	
	Korea	1	110	-	4.231 (1.482–12.079)	0.007		.%			Korea		3	494	Random	2.838 (1.280–6.291)	0.01	69.80%	
	All	10	1274	Fixed	2.762 (1.941–3.942)	0.000		48.40%			All		12	1513	Random	2.345 (1.601–3.435)	0.000	50.50%	0.858
Vascular invasion	China	2	180	Fixed	5.275 (2.287–12.167)	0.000		0.00%		0.142	China		-	-	-	-	-	-	
	Japan	4	618	Fixed	2.002 (1.413–2.836)	0.000		18.40%			Japan		-	-	-	-	-	-	
	Korea	-	-	-	-	-		-			Korea		-	-	-	-	-	-	
	All	6	798	Fixed	2.368 (1.725–3.252)	0.000		43.50%			All		-	-	-	-	-	-	-
Histological differentiation	China	6	703	Fixed	1.684 (1.055–2.686)		0.029		47.80%	0.094	China	11		1164	Fixed	2.035 (1.577–2.627)	0.000	35.70%	
	Japan	4	621	Random	2.941 (1.463–5.914)		0.002		75.50%		Japan	-		-	-	-	-	-	
	Korea	-	-	-	-		-		-		Korea	2		400	Fixed	0.954 (0.594–1.533)	0.846	0.00%	
	All	10	1324	Random	2.112 (1.410–3.163)		0.000		64.10%		All	13		1564	Fixed	1.715 (1.371–2.145)	0.000	48.00%	0.258
Size	China	4	522	Random	2.118 (1.468–3.056)		0.000		74.40%	0.945	China	2		162	Fixed	2.197 (1.071–4.506)	0.032	55.20%	
	Japan	1	152	-	1.404 (0.724–2.722)		0.315		.%		Japan	1		249	-	3.063 (1.685–5.566)	0.000	.%	
	Korea	-	-	-	-		-		-		Korea	1		90	-	1.078 (0.461–2.524)	0.862	.%	
	All	5	674	Random	1.921 (1.395–2.647)		0.000		68.80%		All	4		501	Fixed	2.188 (1.468–3.259)	0.000	50.90%	0.950
Sex	China	3	330	Fixed	1.102 (0.707–1.719)		0.667		44.40%	0.883	China	8		846	Fixed	1.371 (1.005–1.869)	0.046	21.30%	
	Japan	3	406	Random	0.750 (0.499–1.126)		0.165		53.90%		Japan	2		364	Fixed	1.584 (0.903–2.780)	0.109	0.00%	
	Korea	1	114	-	1.043 (0.381–2.853)		0.935		.%		Korea	2		400	Fixed	1.468 (0.877–2.459)	0.144	0.00%	
	All	7	850	Fixed	0.905 (0.679–1.205)		0.495		37.10%		All	12		1610	Fixed	1.431(1.126–1.818)	0.003	0.00%	0.359
Age	China	6	667	Fixed	0.741(0.529–1.040)		0.083		0.00%	0.920	China	7		687	Fixed	1.533 (1.107–2.124)	0.01	57.20%	
	Japan	4	622	Fixed	0.976 (0.686–1.388)		0.891		37.20%		Japan	2		364	Fixed	1.363 (0.803–2.313)	0.251	0.00%	
	Korea	1	114	-	0.855 (0.310–2.360)		0.763		.%		Korea	1		90	-	1.571 (0.670–3.685)	0.299	.%	
	All	11	1403	Fixed	0.846 (0.667–1.072)		0.166		0.00%		All	10		1141	Fixed	1.494 (1.148–1.944)	0.003	36.40%	0.970

NS, number of studies, NP, number of patients; OR, odd ratio; RR, risk ratio; CI, confidence interval.

#### Correlation of HIF-1α expression with 5-year overall survival

**Figure 2:**
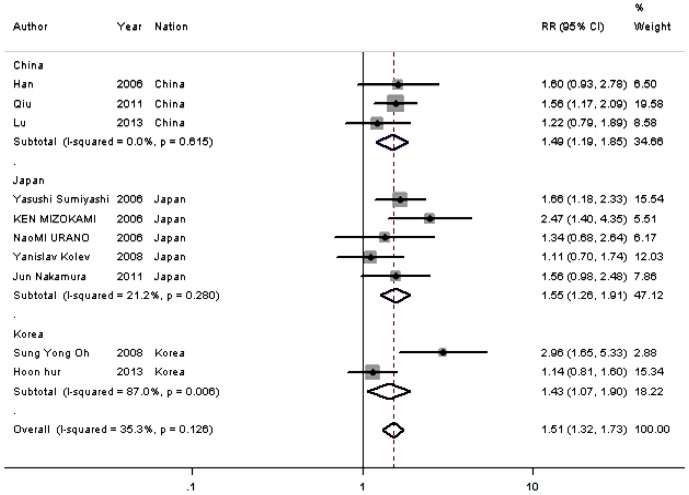
Meta-analysis on the relation between HIF-1α expression and 5-year overall survival (OS). The summary RR and 95% CIs were shown (fixed-effect model analysis).

Meta-analysis on the prognostic value of HIF-1α expression showed that the overall survival rate at 5 years after the initial treatment was significantly lower in cases with overexpressed HIF-1α in 10 studies (1333 patients). The combined RR was 1.508 (95% CI  = 1.318–1.725; P<0.001), with low heterogeneity in the data (I^2^ = 35.3%, subgroup difference P = 0.126) (Fig. 2, Table 2). When stratifying for ethnicity, results were similar among patients from China, Japan, and Korea (Fig. 2, Table 2).

#### Correlation of HIF-1α expression with clinical variables

When stratifying clinicopathological variables by the depth of invasion of gastric cancer, statistical significance was observed. Patients with T_3_ and T_4_ gastric cancer had higher HIF-1α expression in 9 studies (1188 patients; OR  = 3.050, 95% CI  = 2.067–4.501, P<0.001) than those with T_1_ and T_2_ gastric cancer, with moderate between-study heterogeneity (I^2^ = 53.8%, subgroup difference P = 0.027) (Table 2). When stratifying for the lymph node status of gastric cancer, the results showed that overexpressed HIF-1α was significantly associated with lymph node metastasis in 11 studies (1415 patients; OR  = 3.486, 95% CI  = 2.737 –4.440, P<0.001), with low heterogeneity among studies (I^2^ = 31.4%, subgroup difference P = 0.148) (Table 2). When stratifying for vascular invasion status, the overexpression of HIF-1α showed a significant association with the presence of vascular invasion in 6 studies (798 patients; OR  = 2.368, 95% CI  = 1.725–3.252, P<0.001), with low between-study heterogeneity (I^2^ = 43.5%, subgroup difference P = 0.115) (Table 2). When stratifying the distant metastasis of gastric cancer, HIF-1α expression was significantly associated with distant metastasis in 5 studies (469 patients; OR  = 6.635, 95% CI  = 1.855–23.738, P = 0.004), although, with evident between-study heterogeneity (I^2^ = 73.3%, subgroup difference P = 0.005) (Table 2). When further stratifying the TNM stage, HIF-1α expression of patients with stages III and IV gastric cancer was much higher than those with stage I and II gastric cancer in 10 studies (1274 patients; OR  = 2.762, 95% CI  = 1.941–3.942, P<0.001), without significant between-study heterogeneity (I^2^ = 48.4%, subgroup difference P = 0.042) (Table 2).

We also observed a correlation between overexpressed HIF-1α with poor histological differentiation in 10 studies (1324 patients) because the pooled ORs (95% CI) were 2.112 (1.410–3.163, P<0.001) and the tumor size was 1.921 (1.395–2.647, P<0.001), but not for sex (0.905; 0.679–1.205, P = 0.495) and age (0.846; 0.667–1.072, P = 0.166), among all Asian patients (Table 2).

#### Correlation of PTEN expression with prognosis

**Figure 3:**
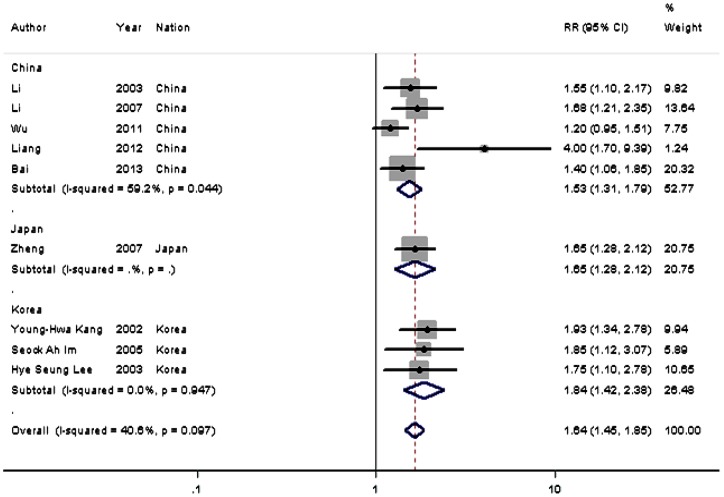
Meta-analysis on the relation between PTEN expression and 5-year overall survival (OS). The summary RR and 95% CIs were shown (fixed-effect model analysis).

The combined results showed that PTEN expression in Asian patients with gastric cancer was significantly lower than controls among 11 studies (1498 patients and 1164 controls; OR  = 16.930, 95% CI  = 8.613–33.280, P<0.001) (Table 2). Reduced PTEN expression correlated with poor overall survival in 9 studies (1551 patients; RR  = 1.637, 95% CI  = 1.452–1.845, P<0.001) (Fig. 3 and Table 2). Subgroup analysis showed a trend that reduced PTEN levels were associated with the following factors: depth of invasion (12 studies, 1599 patients; OR  = 2.604, 95% CI  = 1.554–4.366, P<0.001); lymph node metastasis (18 studies, 2258 patients; OR  = 2.484, 95% CI  = 1.836–3.360, P<0.001); distant metastasis (9 studies, 1393 patients; OR  = 2.528, 95% CI  = 1.703–3.751, P<0.001); TNM stage; 12 studies, 1513 patients; OR  = 2.345, 95% CI  = 1.601–3.435, P<0.001); histological differentiation (13 studies, 1564 patients; OR  = 1.715, 95% CI  = 1.371–2.145, P<0.001); tumor size (4 studies, 501 patients; OR  = 2.188, 95% CI  = 1.468–3.259, P<0.001); sex (12 studies, 1610 patients; OR  = 1.431, 95% CI  = 1.126–1.818, P = 0.003); and age (10 studies, 1141 patients; OR  = 1.494, 95% CI  = 1.148–1.944, P = 0.003) (Table 2).

#### Correlation of CD44v6 expression with prognosis

**Figure 4:**
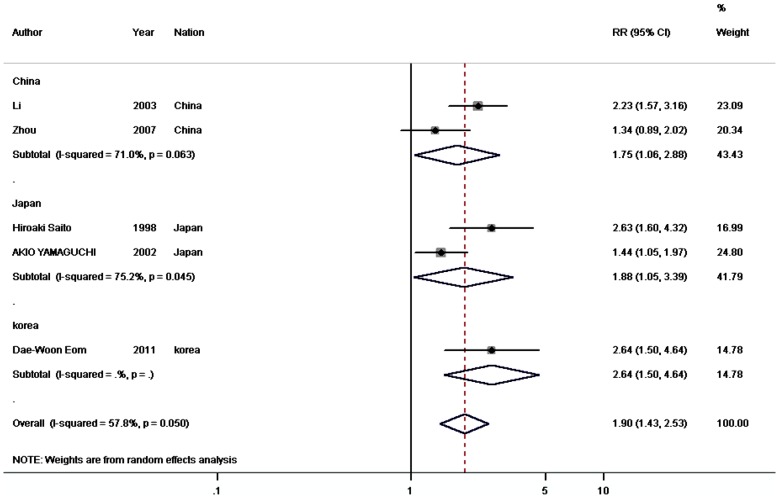
Meta-analysis on the relation between CD44v6 expression and 5-year overall survival (OS). The summary RR and 95% CIs were shown (random-effect model analysis).

A similar result was observed for CD44v6. The pooled analyses of 9 studies showed that CD44v6 expression in gastric cancer (758 patients and 621 controls) was significantly higher compared to controls (OR  = 82.673, 95% CI  = 44.980–151.953, P<0.001) (Table 3). CD44v6 overexpression was associated with a higher risk of death at 5 years in 5 studies (767 patients; RR  = 1.901, 95% CI  = 1.432–2.525, P<0.001) (Fig. 4, Table 3). Of the tumor-related factors, increased depth of invasion (10 studies, 932 patients; OR  = 2.251, 95% CI  = 1.415–3.582, P = 0.001), lymph node metastasis (12 studies, 1149 patients; OR  = 3.027, 95% CI  = 2.313–3.962, P<0.001), distant metastasis (5 studies, 578 patients; OR  = 3.431, 95% CI  = 2.157–5.456, P<0.001), vascular invasion (6 studies, 753 patients; OR  = 1.926, 95% CI  = 1.170–3.171, P = 0.01), histological differentiation (8 studies, 573 patients; OR  = 1.704, 95% CI  = 1.193 2.434, P = 0.003), and TNM stage (7 studies, 589 patients; OR  = 3.918, 95% CI  = 2.658–5.777, P<0.001) provided remarkable prognostic information (Table 3).

**Table 3:** Meta-analyses of Surviving and Cd44v6 expressions on gastric cancer.

Stratification of gastric cancer	Surviving								CD44v6							
	Nation	NS	NP	Model	OR(RR) (95%CI)	P	I^ 2^	P _bias_	Nation	NS	NP	Model	OR(RR) (95%CI)	P	I^ 2^	P_ bias_
Case-Control	China	8	828	Fixed	60.162 (32.857–110.159)	0.000	20.10%	0.011	China	6	715	Fixed	45.271 (21.598 –94.889)	0.000	0.00%	
	Japan	1	348	-	184.406 (11.290–3011.948)	0.000	.%		Japan	2	466	Fixed	220.330 (52.955–916.733)	0.000	0.00%	
	Korea	2	526	Fixed	212.274 (29.119–1547.435)	0.000	0.00%		Korea	1	198	-	82.673 (44.980–151.953)	0.000	.%	
	All	11	1702	Fixed	83.622 (46.476–150.455)	0.000	20.40%		All	9	1379	Fixed	82.673 (44.980–151.953)	0.000	31.50%	0.135
Overall 5-year survival	China	4	371	Fixed	1.731 (1.380–2.172)	0.000	0.00%	0.008	China	2	198	Fixed	1.748 (1.060–2.884)	0.029	71.00%	
	Japan	-	-	-	-	-	-		Japan	2	336	Random	1.884 (1.048–3.386)	0.034	75.20%	
	Korea	2	263	Fixed	1.500 (1.194–1.885)	0.001	0.00%		Korea	1	233	-	2.640 (1.502–4.639)	0.001	.%	
	All	6	634	Fixed	1.627 (1.384–1.913)	0.000	0.00%		All	5	767	Random	1.901 (1.432–2.525)	0.000	57.80%	0.282
The depth of invasion	China	6	584	Fixed	1.663 (0.751–3.682)	0.21	79.70%	0.027	China	6	381	Fixed	3.183 (1.879–5.390)	0.000	9.70%	
	Japan	2	309	Fixed	0.626 (0.378–1.036)	0.069	0.00%		Japan	4	551	Fixed	1.599 (0.887–2.883)	0.119	56.10%	
	Korea	3	1339	Fixed	1.250 (0.387–4.043)	0.709	91.50%		Korea	-	-	-	-	-	-	
	All	12	2232	Fixed	1.292 (0.754–2.214)	0.352	84.30%		All	10	932	Random	2.251 (1.415–3.582)	0.001	51.9%	0.083
Lymph node status	China	8	736	Fixed	1.764 (0.743–4.190)	0.198	83.50%	0.083	China	7	499	Fixed	4.219 (2.807–6.343)	0.000	0.00%	
	Japan	3	438	Fixed	0.963 (0.636–1.459)	0.859	0.00%		Japan	4	551	Fixed	2.182 (1.486–3.205)	0.000	44.10%	
	Korea	3	1339	Fixed	1.245 (0.602–2.572)	0.554	80.40%		Korea	1	99	-	3.676 (1.232–10.963)	0.020	.%	
	All	14	2500	Fixed	1.429 (0.909–2.247)	0.121	80.60%		All	12	1149	Fixed	3.027 (2.313–3.962)	0.000	33.60%	0.045
Distant metastasis	China	3	317	Fixed	2.293 (1.212–4.340)	0.011	62.60%	0.300	China	3	259	Fixed	3.248 (1.654–6.378)	0.001	0.00%	
	Japan	-	-	-	-	-	-		Japan	2	319	Fixed	3.621 (1.918–6.836)	0.000	64.20%	
	Korea	1	106	-	1.000 (0.325–3.079)	1.000	.%		Korea	-	-	-	-	-	-	
	All	4	423	Fixed	1.901 (1.101–3.280)	0.021	53.20%		All	5	578	Fixed	3.431 (2.157–5.456)	0.000	0.00%	0.138
TNM stage	China	5	414	Random	3.206 (1.338–7.680)	0.009	74.10%	0.861	China	5	355	Fixed	5.467 (3.289–9.087)	0.000	23.70%	
	Japan	-	-	-	-	-	-		Japan	1	135	-	2.103 (0.963–4.593)	0.062	.%	
	Korea	1	106	-	3.202 (1.448–7.084)	0.004	.%		Korea	1	99	-	3.063 (1.144–8.201)	0.026	.%	
	All	6	520	Random	3.215 (1.624–6.364)	0.001	67.60%		All	7	589	Fixed	3.918 (2.658–5.777)	0.000	35.80%	0.073
Vascular invasion	China	1	97	-	1.378 (0.499–3.806)	0.536	.%	0.141	China	1	103	-	2.945 (0.996–8.711)	0.051	.%	
	Japan	2	309	Fixed	0.637 (0.382–1.063)	0.085	0.00%		Japan	4	551	Random	1.771 (0.877–3.576)	0.111	69.10%	
	Korea	1	157	-	0.779 (0.292–2.075)	0.617	.%		Korea	1	99	-	1.892 (0.712–5.026)	0.201	.%	
	All	4	563	Fixed	0.753 (0.500–1.134)	0.174	0.00%		All	6	753	Fixed	1.926 (1.170–3.171)	0.010	51.40%	0.632
Histological differentiation	China	8	746	Fixed	0.829 (0.494–1.391)	0.478	61.90%	0.347	China	7	438	Fixed	1.845 (1.217–2.796)	0.004	30.00%	
	Japan	1	135	-	0.256 (0.109–0.600)	0.002	.%		Japan	1	135	-	1.362 (0.678–2.736)	0.386	.%	
	Korea	1	157	-	0.796 (0.418–1.515)	0.487	.%		Korea	-	-	-	-	-	-	
	All	10	1038	Fixed	0.730 (0.460–1.158)	0.181	65.00%		All	8	573	Fixed	1.704 (1.193–2.434)	0.003	22.9%	0.534
Size	China	4	438	Fixed	1.049 (0.713–1.544)	0.808	0.00%	0.635	China	-	-	-	-	-	-	
	Japan	-	-	-	-	-	-		Japan	-	-	-	-	-	-	
	Korea	2	263	Fixed	2.876 (1.702–4.860)	0.000	0.00%		Korea	-	-	-	-	-	-	
	All	6	701	Fixed	1.508 (1.110–2.048)	0.009	49.20%		All	-	-	-	-	-	-	-
Sex	China	7	688	Fixed	1.029 (0.735–1.441)	0.868	0.00%	0.548	China	3	147	Fixed	0.737 (0.343–1.582)	0.433	15.50%	
	Japan	2	252	Fixed	1.985 (1.118–3.522)	0.019	34.70%		Japan	2	223	Fixed	0.827 (0.459–1.489)	0.526	0.00%	
	Korea	2	263	Fixed	0.815 (0.486–1.367)	0.439	26.50%		Korea	-	-	-	-	-	-	
	All	11	1203	Fixed	1.103(0.856–1.420)	0.448	8.00%		All	5	370	Fixed	0.792 (0.497–1.263)	0.327	0.00%	0.909
Age	China	5	487	Fixed	0.797 (0.550–1.156)	0.232	0.00%	0.778	China	3	144	Fixed	1.478 (0.685–3.187)	0.319	0.00%	
	Japan	3	426	Fixed	1.013 (0.668–1.535)	0.952	0.00%		Japan	1	135	-	0.948 (0.471–1.906)	0.880	.%	
	Korea	2	263	Fixed	1.291 (0.787–2.115)	0.312	0.00%		Korea	-	-	-	-	-	-	
	All	10	1176	Fixed	0.970 (0.762–1.234)	0.803	0.00%		All	4	279	Fixed	1.160 (0.693–1.940)	0.572	0.00%	0.433

NS, number of studies, NP, number of patients; OR, odd ratio; RR, risk ratio; CI, confidence interval.

#### Correlation of Survivin expression with prognosis

**Figure 5:**
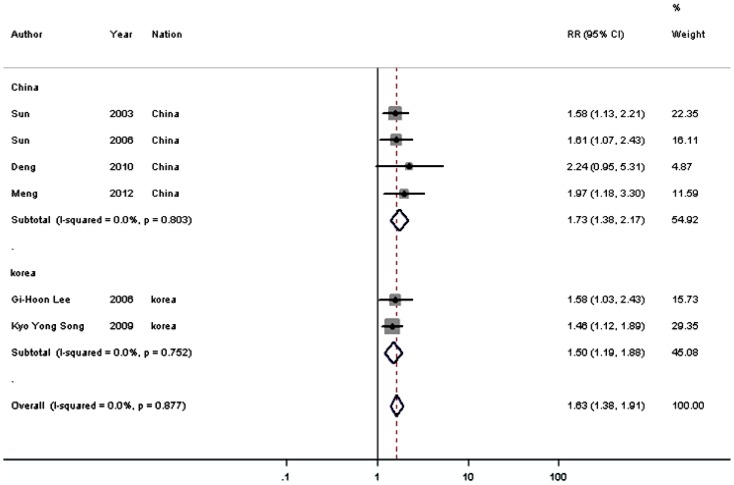
Meta-analysis on the relation between Survivin expression and 5-year overall survival (OS). The summary RR and 95% CIs were shown (fixed-effect model analysis).

Compared to normal controls, the overexpression of Survivin was associated with worse outcome in gastric cancer among 11 studies (863 patients and 839 controls; OR  = 83.622, 95% CI  = 46.476–150.455, P<0.001) (Table 3). This result from the pooled estimate was statistically significant for detrimental 5-year overall survival in 6 studies (634 patients; RR  = 1.627, 95% CI  = 1.384–1.913, P<0.001) (Fig. 5, Table 3). Reduced survival was heavily influenced by tumor size (6 studies, 701 patients; OR  = 1.508, 95% CI  = 1.110–2.048, P  = 0.009), distant metastasis (4 studies, 423 patients; OR  = 1.901, 95% CI  = 1.101–3.280, P = 0.021), and TNM stage (6 studies, 520 patients; OR  = 3.215, 95% CI  = 1.624–6.364, P  = 0.001) (Table 3).

### Sensitivity analysis and publication bias

In the present study, sensitivity analysis indicated that the pooled RR (or OR) was not significantly influenced by omitting any single study at a given time. In addition, the results showed there was no evidence of publication bias for most subgroup analyses (Table 2 and 3). However, the potential biases observed for depth of invasion for the HIF-1α group (P_bias_  = 0.017), case-control study (P_bias_  = 0.034) and overall survival (P_bias_  = 0.006) for the PTEN group, and case-control study (P_bias_  = 0.011), depth of invasion (P_bias_  = 0.027), and overall survival (P_bias_  = 0.008) for the Survivin group could be ruled out by Begg's and Egger's tests (Table 2 and 3).

## Discussion

Almost two-thirds of the world's gastric cancer cases occur in Asia (China, Japan, and Korea) [15]. The mortality of cancer patients is largely caused by metastases rather than their primary tumors at the time of diagnosis. Therefore, identifying the risk of disease recurrence and mortality in gastric cancer patients is critical to monitor patients and select appropriate adjunctive therapies in clinical practice [16], [17]. However, useful biomarkers for predicting the prognosis of gastric cancer patients have not been well studied. Here, we introduced one subset of potential clinically useful biomarkers, HIF-1a/PTEN/CD44v6/Survivin, and precisely estimated their prognosis and clinicopathological significance.

Mounting evidence suggests that hypoxic tumor microenvironments, especially the overexpression of HIF-1a, are strongly implicated as the hallmark of a wide variety of human malignancies [18], [19]. When activated by the novel tumor suppressor gene PTEN [5], [20], HIF-1α can transcriptionally regulate a host of hypoxia-responsive molecules that contribute to drug resistance, epithelial-mesenchymal transition, survival, angiogenesis, and metastasis [9], [10], [21], [22], [23], including inducers of angiogenesis (e.g., VEGF), proliferation of regulatory proteins (e.g., Survivin), and mediators of metastasis (e.g., CD44v6, MMP, E-cadherin). In this study, we found that the overexpression of HIF-1α occurred at a median frequency of 54.1% in gastric cancer. Patients who expressed high levels of HIF-1α were associated with a worse outcome, with a pooled risk for overall survival (RR  = 1.508) that was similar to that obtained in a recently published study on hepatocellular carcinoma (HR  = 1.65) [24]. Moreover, aberrant expression of PTEN, CD44v6, and Survivin were also observed in tumors correlating with poor overall survival, with risk of death at 5 years of 1.637, 1.901, and 1.627, respectively (Table 2 and 3). Subgroup analysis confirmed that the reduced survival was significantly correlated with increased dedifferentiation, tumor size, advanced tumor invasion, lymph node spread, distant metastasis, vascular invasion, and higher TNM stage, indicating increased biological aggressiveness and a greater possibility of systemic diffusion.

Gastric tumors can trigger the substantial development of new blood vessels for tumor growth, maintenance, and metastasis [25], [26]. The high proliferation of tumor cells can induce local hypoxia, which is a strong stimulus for HIF-1α. Loss of PTEN in gastric cancer promotes tumor angiogenesis and invasion by increasing expression of VEGF through the increase of the HIF-1α protein level, which is an active process that requires the degradation of the extracellular matrix, the increase of microvascular permeability both in the blood and lymphatic vessels, and interstitial fluid pressure (IFP) [27], favoring the progression of the intravasation and extravasation of tumor cells. This may offer a possible explanation for the observed strong statistical association of HIF-1α overexpression with advanced tumor invasion, lymph node spread, vascular invasion, and distant metastasis. Recent studies have shown that the percentage of CD44 positive cells expressing variant exons v6 (CD44v6) in tumor cells could be significantly increased by HIF-1α-mediated transcription under hypoxic conditions [28]. CD44v6, one cell adhesion molecule, has been proposed to function in the homing of lymphocytes, extracellular matrix binding, cell migration, and invasion. Therefore, a high level of CD44v6 expression may also contribute to the aggressive phenotype. In addition, the upregulation of Survivin by HIF-1α and PTEN contributed to cisplatin (CDDP) resistance, indicating that inhibition of these pathways may be a potential strategy for overcoming CDDP resistance in the treatment of gastric cancer [29].

**Figure 6:**
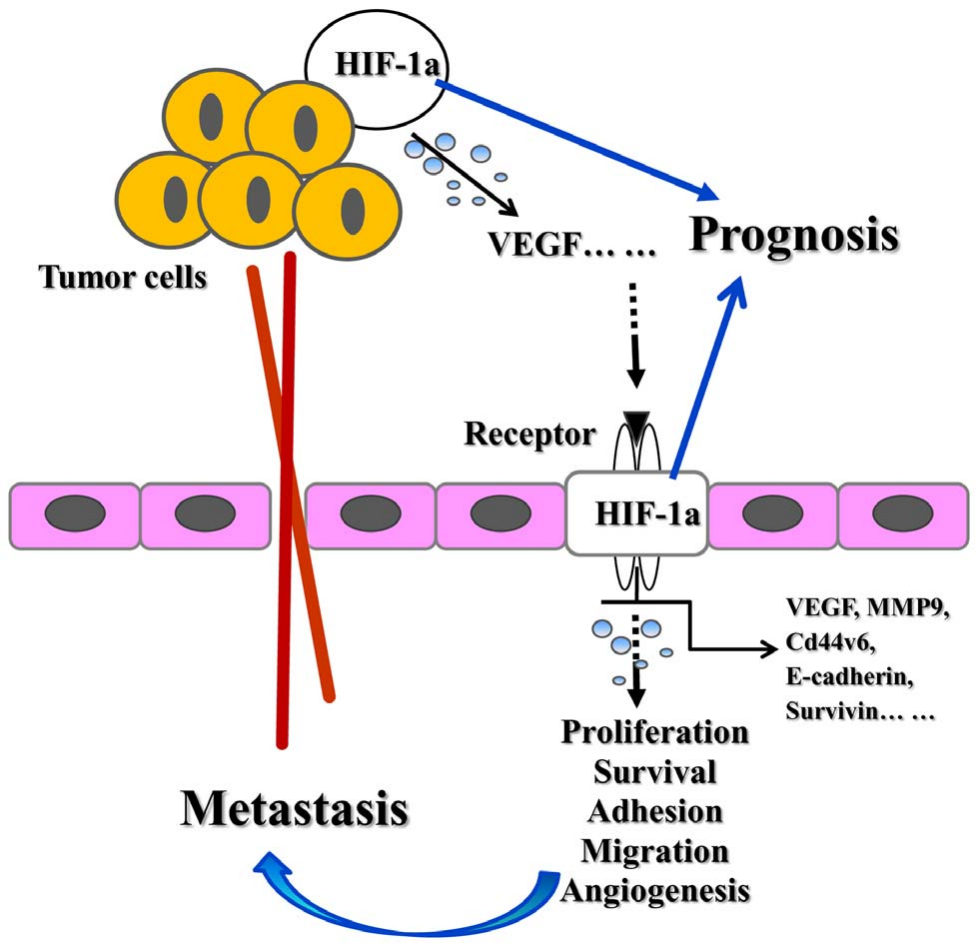
Hypoxia regulation of the metastasis cascade.

These analyses have several important implications. First, we show that the abnormal expression of HIF-1α/PTEN/CD44v6/Survivin is associated with worse outcome, which suggests that each protein may be a useful therapeutic target for drug development. Currently, some clinical trials targeting HIF-1α/PTEN/Survivin at different phases are being developed, which will likely benefit populations with certain conditions (http://www.clinicaltrials.gov) (Table 4). Second, the analyses highlight the importance of developing multiple biomarkers for monitoring treatment response, clinical uses of HIF-1α inhibitors, and prognosis assessment. We have also noticed several new-ly-publicated meta analyses of estimating prognostic value of either HIF-1α or Survivin on gastric cancer patient [30], [31], [32], [33]. Among them, three quarters of electronic databases were used to identify published studies before December, 2012, with the combined HR <2. Because hypoxia is likely to have complex, and even opposing, effects during different stages of tumor development [5], [8], any single molecule cannot be used to independently predict the full prognosis of patients. Combinations of proteins involved in HIF-1α regulation of the metastasis cascade should provide increased prognostic power over individual markers themselves. Moreover, systematically assessing the main prognostic factors in gastric cancer, both tumor- and patient-related, may also have meaningful impacts at the time of diagnosis or surgical treatment, including the depth of tumor invasion, lymph node spread, venous invasion, TNM stage, differentiation, tumor size, as well as sex and age. In present study, the most key results of the meta-analysis of clinical variables among Asian patients showed a magnitude of effect size of OR >2, and in some cases >3. As a rule of the thumb, a prognostic factor with RR (or OR) >2 is considered to be of useful practical value [34]. Therefore, we believe our results will provide more useful and precise information for clinical decision-making regarding gastric cancer. Third, as shown by our previous reports, VEGF appears to be a significant prognostic factor for hematogenous metastasis of gastric cancer (RR  = 2.45, P = 0.000) [34], [35]. In addition, we have proposed three other genes, E-cadherin, Stat3, and MMP-9, as prognosis biomarkers of tumor metastasis. Therefore, analyses combining previous results may show a possible axis of action by HIF-1α and its oncogenic signaling pathway (Fig. 6), which could contribute to improvements in prognosis assessment, functional analysis, and drug-targeted therapy in the prevention and treatment of gastric cancer. From this perspective, we believe that our meta-analysis does indeed present positive significance and novelty.

**Table 4:** Ongoing studies evaluating anti- HIF-1α/PTEN/Survivin therapeutic strategies.

	Study/sponsor	Phase/setting	Experimental arm(s)
Anti- HIF-1α	NCT01120288	Liver Metastases; Phase 1;Active, not recruiting	EZN-2968
	NCT01763931	Newly Diagnosed Operable Breast Cancer; Phase 2; Recruiting	Digoxin
	NCT00522652	Advanced Solid Tumors; Lymphoma; Phase 1; Completed	PX-478
	NCT01047293	Colorectal Carcinoma; Phase 1–2; Recruiting	RAD001; FOLFOX; Bevacizumab
	NCT00117013	Refractory Advanced Solid Neoplasms Expressing HIF-1a; Phase 1; Completed	Topotecan; Fluorine-19-Fluoroded Xyglucose
	NCT00880672	Benign Prostatic Hyperplasia; Phase 4; Completed	Dutasteride
	NCT01251926	Refractory Solid Tumors; Phase 1; Active, not recruiting	EZN-2208; Bevacizumab
	NCT01206764	Advanced Renal Cell Carcinoma; Phase 4; Recruiting	RAD001
	NCT01814449	Human Breast Cancer; Recruiting	18FMISO PET/CT scan; Letrozole
	NCT00389805	Advanced Non-Small Cell Lung Cancer or Other Solid Tumors; Phase 1–2; Completed	bortezomib;pemetrexed disodium
	MD Anderson Cancer Center	Advanced malignancies; Phase I; Completed	bevacizumab and temsirolimus plus liposomal doxorubicin
Anti-PTEN	NCT01283035	Recurrent Platinum-Resistant Ovarian, Fallopian Tube, or Peritoneal Cancer; Phase 2; Recruiting	MK2206
	NCT00490139	Breast Cancer; Phase 3; Recruiting	Lapatinib;Trastuzumab
	NCT00499603	Breast Cancer; Phase 2; Completed	Paclitaxel;5-Fluorouracil;Epirubicin;Cyclophosphamide;RAD001
	NCT01042925	Breast Cancer; Phase 1–2; Completed	XL147 (SAR245408); paclitaxel
	NCT01013324	Endometrial Cancer; Phase 2; Completed	XL147 (SAR245408)
	NCT00387894	Recurrent Glioblastoma Multiforme or Gliosarcoma; Phase 2; Completed	Bevacizumab; Erlotinib
	NCT01550380	Advanced, Metastatic, or Recurrent Endometrial Cancer; Phase 2; Not yet recruiting	BKM120
	NCT00301418	Recurrent/Residual Glioblastoma Multiforme and Anaplastic Astrocytoma; Phase 1–2; Recruiting	Erlotinib
	NCT00895960	Glioblastoma; CNS Disease; Brain Diseases; Phase 1–2; Active, not recruiting	Dasatinib; RT (Radiotherapy); TMZ (Temozolomide)
Anti-Survivin	NCT01088035	Ependymoma; Phase 2; Recruiting	Carboplatin
	NCT00537121	Esophageal Cancer; Gastric Cancer; Liver Cancer; Phase 1	Vorinostat, Irinotecan, Fluorouracil, Leucovorin

There are also limitations that should be noted based on the present analysis. First, because this is a literature-based analysis from which predominantly positive results were published, our estimate for the association between HIF-1α/PTEN/CD44v6/Survivin and poor outcome might be inflated. Therefore, the discrepancies in the conclusions of various studies encouraged researchers to publish their data regardless of the significance of their results, which may limit the publication bias. In the present study, we placed emphasis on assessing biases across studies and pinpointing any potential sources of heterogeneity. Subgroup analyses by ethnicity and clinical variables were also performed. We comprehensively assessed the publication biases by using Begg's and Egger's tests and did not detect any significant deviation among most studies, except for the several factors mentioned in the “Results” section. In view of this, we are confident that the results of our meta-analysis are reliable. In addition, we could not pool the hazard ratios of death because of a lack of time-to-death data. We only figured out the risk ratio of death at fixed time points. Although this measure is less robust because it does not consider the duration of survival until death, this is the only feasible method of the data available [36].

In conclusion, our analyses show that the aberrant expression of HIF-1α, PTEN, CD44v6, and Survivin, as measured by IHC, may predict the 5-year overall survival risk and potential for invasion and metastasis in gastric cancer patients, particularly in Asian patients. These data suggest that the development of strategies against this subset of proteins could lead to a reasonable therapeutic treatment program for gastric cancer. However, further large sample and non-Asian population-based studies are required.

## Supporting Information

Table S1 **Main characteristics of protein expressions on prognostic factors.**
(DOC)

Reference S1 **Supplementary References Enrolled in the Meta-Analyses.**
(DOC)

Checklist S1 **PRISMA 2009 Checklist for the Meta-Analyses.**
(DOC)

Flow Diagram S1 **PRISMA 2009 Flow Diagram for the Meta-Analyses.**
(DOC)
