# Supplementary material for: Clinical and Prognostic Significance of HIF-1α, PTEN, CD44v6, and Survivin for Gastric Cancer: A Meta-Analysis
Source: PLoS One. 2014 Mar 19;9(3):e91842. doi: 10.1371/journal.pone.0091842 (PMC3960154; doi:10.1371/journal.pone.0091842)
Supplement: Table S1 — Main characteristics of protein expressions on prognostic factors. (DOC) [file pone.0091842.s001.doc]

**Supplementary Table 1 Main characteristics of protein expressions** on prognostic factors

|  |  | | **HIF-1a** | |  | **PTEN** | |  | **Survivin** | |  | **CD44v6** | |
| --- | --- | --- | --- | --- | --- | --- | --- | --- | --- | --- | --- | --- | --- |
| **Stratification** | **Type** | **Total** | | **Number** |  | **Total** | **Number** |  | **Total** | **Number** |  | **Total** | **Number** |
| Case-Control | carcinomas | 1821 | | 923 |  | 2662 | 1498 |  | 1702 | 863 |  | 1379 | 758 |
|  | Non-neoplastic mucosa |  |  | 898 |  |  | 1164 |  |  | 839 |  |  | 621 |
| Overall 5-year survival | Mortal | 1333 | | 551 |  | 1551 | 680 |  | 634 | 348 |  | 767 | 441 |
|  | Survival |  |  | 782 |  |  | 871 |  |  | 286 |  |  | 326 |
| The depth of invasion | T3+T4 | 1188 | | 643 |  | 1599 | 1089 |  | 2232 | 1515 |  | 932 | 611 |
|  | T1+T2 |  |  | 545 |  |  | 470 |  |  | 717 |  |  | 321 |
| Lymph node status | Positive | 1415 | | 901 |  | 2258 | 1374 |  | 2500 | 1541 |  | 1149 | 683 |
|  | Negative |  |  | 514 |  |  | 884 |  |  | 959 |  |  | 466 |
| Distant metastasis | Positive | 469 | | 140 |  | 1393 | 136 |  | 423 | 89 |  | 578 | 147 |
|  | Negative |  |  | 329 |  |  | 1257 |  |  | 334 |  |  | 431 |
| TNM stage | III+IV | 1274 | | 620 |  | 1513 | 889 |  | 520 | 273 |  | 589 | 299 |
|  | I+II |  |  | 654 |  |  | 624 |  |  | 247 |  |  | 290 |
| Venous invasion | Positive | 798 | | 346 |  | 623 | 257 |  | 563 | 228 |  | 753 | 313 |
|  | Negative |  |  | 452 |  |  | 366 |  |  | 335 |  |  | 440 |
| Histological differentiation | Pooly | 1324 | | 739 |  | 1564 | 982 |  | 1038 | 590 |  | 573 | 300 |
|  | Well/moderate |  |  | 585 |  |  | 582 |  |  | 448 |  |  | 273 |
| Size | ≥5cm | 674 | | 377 |  | 501 | 255 |  | 701 | 346 |  | - | - |
|  | <5cm |  |  | 297 |  |  | 246 |  |  | 355 |  |  | - |
| Sex | Male | 1403 | | 939 |  | 1610 | 1115 |  | 1203 | 824 |  | 370 | 249 |
|  | Female |  |  | 464 |  |  | 495 |  |  | 379 |  |  | 121 |
| Age | >60 | 850 | | 428 |  | 1141 | 715 |  | 1176 | 584 |  | 279 | 167 |
|  | ≤60 |  |  | 422 |  |  | 426 |  |  | 592 |  |  | 112 |
