# Supplementary material for: Clinical and Prognostic Significance of HIF-1α, PTEN, CD44v6, and Survivin for Gastric Cancer: A Meta-Analysis
Source: PLoS One. 2014 Mar 19;9(3):e91842. doi: 10.1371/journal.pone.0091842 (PMC3960154; doi:10.1371/journal.pone.0091842)
Supplement: Reference S1 — Supplementary References Enrolled in the Meta-Analyses. (DOC) [file pone.0091842.s002.doc]

**Supplementary References Enrolled in the Meta-Analyses of HIF-1α:**

1. Liu Y, Lu MZ, Yuan S. Detection of the expression of hypoxia-inducible factor in gastric mucosal disease by using tissue chip technique. Chinese Journal of General Surgery. 2004; 13: 272-5.

2. Chen WT, Huang CJ, Wu MT, Yang SF, Su YC, Chai CY. Hypoxia-inducible factor-1alpha is associated with risk of aggressive behavior and tumor angiogenesis in gastrointestinal stromal tumor. Jpn J Clin Oncol. 2005;35:207-13.

3. Han B, Xu RH, Shi YX, Li YH, Luo HY, Wang F, et al. [Expression and clinical significance of hypoxia-inducible factor-1alpha in gastric cancer]. Ai Zheng. 2006;25:1439-42.

4. Ru GQ, Zhao ZS, Tang QL, Xu WJ. [Expressions of hypoxia inducible factor-1alpha and insulin-like growth factor-II in gastric carcinoma: correlation with angiogenesis and prognosis]. Zhonghua Wai Ke Za Zhi. 2007;45:905-8.

5. Wu Q, Li ZS, Li DW, Peng ZH, Yang ZR. [Relationship between the expression of hypoxia-inducible factor-1alpha and chemotherapy response in gastric carcinoma]. Zhonghua Wei Chang Wai Ke Za Zhi. 2009;12:498-501.

6. Qiu MZ, Han B, Luo HY, Zhou ZW, Wang ZQ, Wang FH, et al. Expressions of hypoxia-inducible factor-1alpha and hexokinase-II in gastric adenocarcinoma: the impact on prognosis and correlation to clinicopathologic features. Tumour Biol. 2011;32:159-66.

7. Wang Y, Li Z, Zhang H, Jin H, Sun L, Dong H, et al. HIF-1alpha and HIF-2alpha correlate with migration and invasion in gastric cancer. Cancer Biol Ther. 2010;10:376-82.

8. Jia YF, Xiao DJ, Ma XL, Song YY, Hu R, Kong Y, et al. Differentiated embryonic chondrocyte-expressed gene 1 is associated with hypoxia-inducible factor 1alpha and Ki67 in human gastric cancer. Diagn Pathol. 2013;8:37.

9. Lu XX, Chen YT, Feng B, Mao XB, Yu B, Chu XY. Expression and clinical significance of CD73 and hypoxia-inducible factor-1alpha in gastric carcinoma. World J Gastroenterol. 2013;19:1912-8.

10. Mizokami K, Kakeji Y, Oda S, Irie K, Yonemura T, Konishi F, et al. Clinicopathologic significance of hypoxia-inducible factor 1alpha overexpression in gastric carcinomas. J Surg Oncol. 2006;94:149-54.

11. Sumiyoshi Y, Kakeji Y, Egashira A, Mizokami K, Orita H, Maehara Y. Overexpression of hypoxia-inducible factor 1alpha and p53 is a marker for an unfavorable prognosis in gastric cancer. Clin Cancer Res. 2006;12:5112-7.

12. Urano N, Fujiwara Y, Doki Y, Tsujie M, Yamamoto H, Miyata H, et al. Overexpression of hypoxia-inducible factor-1 alpha in gastric adenocarcinoma. Gastric Cancer. 2006;9:44-9.

13. Kolev Y, Uetake H, Takagi Y, Sugihara K. Lactate dehydrogenase-5 (LDH-5) expression in human gastric cancer: association with hypoxia-inducible factor (HIF-1alpha) pathway, angiogenic factors production and poor prognosis. Ann Surg Oncol. 2008;15:2336-44.

14. Nakamura J, Kitajima Y, Kai K, Mitsuno M, Ide T, Hashiguchi K, et al. Hypoxia-inducible factor-1alpha expression predicts the response to 5-fluorouracil-based adjuvant chemotherapy in advanced gastric cancer. Oncol Rep. 2009;22:693-9.

15. Isobe T, Aoyagi K, Koufuji K, Shirouzu K, Kawahara A, Taira T, et al. Clinicopathological significance of hypoxia-inducible factor-1 alpha (HIF-1alpha) expression in gastric cancer. Int J Clin Oncol. 2013;18:293-304.

16. Oh SY, Kwon HC, Kim SH, Jang JS, Kim MC, Kim KH, et al. Clinicopathologic significance of HIF-1alpha, p53, and VEGF expression and preoperative serum VEGF level in gastric cancer. BMC Cancer. 2008;8:123.

17. Hur H, Xuan Y, Kim YB, Lee G, Shim W, Yun J, et al. Expression of pyruvate dehydrogenase kinase-1 in gastric cancer as a potential therapeutic target. Int J Oncol. 2013;42:44-54.

**Supplementary References Enrolled in the Meta-Analyses of PTEN:**

1. Li XL, Wang YP, Wu DY, Zhang SM, Xin Y. Expression of PTEN, E-cadherin and 67KD-LR in gastric cancer: correlation with migration and invasion. Natl Med J China. 2003; 83: 599-601.

2. Yang L, Kuang LG, Zheng HC, Li JY, Wu DY, Zhang SM, et al. PTEN encoding product: a marker for tumorigenesis and progression of gastric carcinoma. World J Gastroenterol. 2003;9:35-9.

3. Zheng HC, Li YL, Sun JM, Yang XF, Li XH, Jiang WG, et al. Growth, invasion, metastasis, differentiation, angiogenesis and apoptosis of gastric cancer regulated by expression of PTEN encoding products. World J Gastroenterol. 2003;9:1662-6.

4. Li JY, Zheng HC, Yang L, Xu L, Yang XF, Gao H, et al. [Altered expression of PTEN gene and LOH of its epigenetic microsatellite in gastric carcinoma]. Zhonghua Zhong Liu Za Zhi. 2004;26:389-92.

5. Deng H, Wu RL, Zhou HY, Huang X, Chen Y, Liu LJ. Significance of Survivin and PTEN expression in full lymph node-examined gastric cancer. World J Gastroenterol. 2006;12:1013-7.

6 Li DW, Wu Q, Peng ZH, Yang ZR, Wang Y. [Expression and significance of Notch1 and PTEN in gastric cancer]. Ai Zheng. 2007;26:1183-7.

7. Wang Z, Wang XC, Li JF, Sun XC, Wang X. The expression significance and associativity of ILK and PTEN in gastric cancer tissue. Chinese German Journal of Clinical Oncology. 2008; 7: 701-3.

8. Guo CY, Xu XF, Wu JY, Liu SF. PCR-SSCP-DNA sequencing method in detecting PTEN gene mutation and its significance in human gastric cancer. World J Gastroenterol. 2008;14:3804-11.

9. Wu Q, Yang Z, Hu S, Su T, An Y, Zhang Z, et al. Stem cell associated genes working with one miRNA cluster have different clinic pathologic values in gastric cancer. Pathol Oncol Res. 2011;17:939-46.

10. Liang YM, Li XH, Li WM, Lu YY. Prognostic significance of PTEN, Ki-67 and CD44s expression patterns in gastrointestinal stromal tumors. World J Gastroenterol. 2012;18:1664-71.

11. Yang Z, Yuan XG, Chen J, Luo SW, Luo ZJ, Lu NH. Reduced expression of PTEN and increased PTEN phosphorylation at residue Ser380 in gastric cancer tissues: a novel mechanism of PTEN inactivation. Clin Res Hepatol Gastroenterol. 2013;37:72-9.

12. Li M, Sun H, Song L, Gao X, Chang W, Qin X. Immunohistochemical expression of mTOR negatively correlates with PTEN expression in gastric carcinoma. Oncol Lett. 2012;4:1213-8.

13. Bai ZG, Ye YJ, Shen DH, Lu YY, Zhang ZT, Wang S. PTEN expression and suppression of proliferation are associated with Cdx2 overexpression in gastric cancer cells. Int J Oncol. 2013;42:1682-91.

14. Zhu X, Qin X, Fei M, Hou W, Greshock J, Bachman KE, et al. Loss and reduced expression of PTEN correlate with advanced-stage gastric carcinoma. Exp Ther Med. 2013;5:57-64.

15. Zheng H, Takahashi H, Murai Y, Cui Z, Nomoto K, Tsuneyama K, et al. Low expression of FHIT and PTEN correlates with malignancy of gastric carcinomas: tissue-array findings. Appl Immunohistochem Mol Morphol. 2007;15:432-40.

16. Hino R, Uozaki H, Murakami N, Ushiku T, Shinozaki A, Ishikawa S, et al. Activation of DNA methyltransferase 1 by EBV latent membrane protein 2A leads to promoter hypermethylation of PTEN gene in gastric carcinoma. Cancer Res. 2009;69:2766-74.

17. Kang YH, Lee HS, Kim WH. Promoter methylation and silencing of PTEN in gastric carcinoma. Lab Invest. 2002;82:285-91.

18. Im SA, Lee KE, Nam E, Kim DY, Lee JH, Han HS, et al. Potential prognostic significance of p185(HER2) overexpression with loss of PTEN expression in gastric carcinomas. Tumori. 2005;91:513-21.

19. Lee HS, Lee HK, Kim HS, Yang HK, Kim WH. Tumour suppressor gene expression correlates with gastric cancer prognosis. J Pathol. 2003;200:39-46.

20. Park GS, Joo YE, Kim HS, Choi SK, Rew JS, Park CS, et al. [Expression of PTEN and its correlation with angiogenesis in gastric carcinoma]. Korean J Gastroenterol. 2005;46:196-203.

**Supplementary References Enrolled in the Meta-Analyses of Survivin:**

1. Yu J, Leung WK, Ebert MP, Ng EK, Go MY, Wang HB, et al. Increased expression of survivin in gastric cancer patients and in first degree relatives. Br J Cancer. 2002;87:91-7.

2. Zhu XD, Lin GJ, Qian LP, Chen ZQ. Expression of survivin in human gastric carcinoma and gastric carcinoma model of rats. World J Gastroenterol. 2003;9:1435-8.

3. Sun YH, Wang XF, Qin XY, Wang CP, Lu SH, Zhou YN. Expression and clinical significance of survivin in gastric cancer. Chin J Gastrointest Surg. 2003; 6: 191-4.

4. Yao XQ, Liu FK, Qi XP, Wu B, Yin HL, Ma HH, et al. [Expression of survivin in human gastric adenocarcinomas: correlation with proliferation and apoptosis]. Zhonghua Wai Ke Za Zhi. 2004;42:145-8.

5. Li YH, Wang C, Meng K, Chen LB, Zhou XJ. Influence of survivin and caspase-3 on cell apoptosis and prognosis in gastric carcinoma. World J Gastroenterol. 2004;10:1984-8.

6. Lu XY, Liu NZ. Expression of Survivin Gene and Its Relationship with Expression of caspase-3, p53 in Gastric Carcinomas. J Huazhong Univ Sci Tech. 2004; 33: 724-33.

7. Deng H, Wu RL, Zhou HY, Huang X, Chen Y, Liu LJ. Significance of Survivin and PTEN expression in full lymph node-examined gastric cancer. World J Gastroenterol. 2006;12:1013-7.

8. Sun YS, Ye ZY, Zhao ZS, Shi D, Zou SC. [Expression of vascular endothelial growth factor C and survivin in gastric carcinoma and their clinical implications]. Zhonghua Wei Chang Wai Ke Za Zhi. 2006;9:264-7.

9. Cheng Z, Hu L, Fu W, Zhang Q, Liao X. Expression of survivin and its splice variants in gastric cancer. J Huazhong Univ Sci Technolog Med Sci. 2007;27:393-8.

10. Han JC, Zhang KL, Chen XY, Jiang HF, Kong QY, Sun Y, et al. Expression of seven gastric cancer-associated genes and its relevance for Wnt, NF-kappaB and Stat3 signaling. APMIS. 2007;115:1331-43.

11. Deng JY, Sun D, Liu XY, Pan Y, Liang H. STAT-3 correlates with lymph node metastasis and cell survival in gastric cancer. World J Gastroenterol. 2010;16:5380-7.

12. Li Y, Tan BB, Fan LQ, Zhao Q, Liu Y, Wang D. Expression of COX-2, survivin in regional lymph node metastases of gastric carcinoma and the correlation with prognosis. Hepatogastroenterology. 2010;57:1435-41.

13. Meng JR, Tang HZ, Zhou KZ, Shen WH, Guo HY. TFF3 and survivin expressions associate with a lower survival rate in gastric cancer. Clin Exp Med. 2012.

14. Deng H, Zhen H, Fu Z, Huang X, Zhou H, Liu L. The antagonistic effect between STAT1 and Survivin and its clinical significance in gastric cancer. Oncol Lett. 2012;3:193-9.

15. Lu CD, Altieri DC, Tanigawa N. Expression of a novel antiapoptosis gene, survivin, correlated with tumor cell apoptosis and p53 accumulation in gastric carcinomas. Cancer Res. 1998;58:1808-12.

16. Hino R, Uozaki H, Inoue Y, Shintani Y, Ushiku T, Sakatani T, et al. Survival advantage of EBV-associated gastric carcinoma: survivin up-regulation by viral latent membrane protein 2A. Cancer Res. 2008;68:1427-35.

17. Okayama H, Kumamoto K, Saitou K, Hayase S, Kofunato Y, Sato Y, et al. CD44v6, MMP-7 and nuclear Cdx2 are significant biomarkers for prediction of lymph node metastasis in primary gastric cancer. Oncol Rep. 2009;22:745-55.

18. Lee GH, Joo YE, Koh YS, Chung IJ, Park YK, Lee JH, et al. Expression of survivin in gastric cancer and its relationship with tumor angiogenesis. Eur J Gastroenterol Hepatol. 2006;18:957-63.

19. Song KY, Jung CK, Park WS, Park CH. Expression of the antiapoptosis gene Survivin predicts poor prognosis of stage III gastric adenocarcinoma. Jpn J Clin Oncol. 2009;39:290-6.

20. Kim MA, Lee HE, Lee HS, Yang HK, Kim WH. Expression of apoptosis-related proteins and its clinical implication in surgically resected gastric carcinoma. Virchows Arch. 2011;459:503-10.

**Supplementary References Enrolled in the Meta-Analyses of CD44v6:**

1. Xin Y, Grace A, Gallagher MM, Curran BT, Leader MB, Kay EW. CD44V6 in gastric carcinoma: a marker of tumor progression. Appl Immunohistochem Mol Morphol. 2001;9:138-42.

2. Li Y, Zhang JH, Kuang G, Yang JQ, Zhao Q, Wang XL, et al. [Expression of MUC1, CD44v6, nm23 in gastric carcinomas and regional lymph node tissues and their association with invasion, metastasis, and prognosis of the tumor]. Ai Zheng. 2003;22:985-9.

3. Chen JQ, Zhan WH, He YL, Peng JS, Wang JP, Cai SR, et al. Expression of heparanase gene, CD44v6, MMP-7 and nm23 protein and their relationship with the invasion and metastasis of gastric carcinomas. World J Gastroenterol. 2004;10:776-82.

4. Zhao ZQ, Yu PW, Zhao YL, Shi Y. [Expressions of RhoC, CD44v6 and ICAM-1 in human gastric cancer and its clinicopathological significance]. Zhonghua Wei Chang Wai Ke Za Zhi. 2005;8:352-5.

5. Liu YJ, Yan PS, Li J, Jia JF. Expression and significance of CD44s, CD44v6, and nm23 mRNA in human cancer. World J Gastroenterol. 2005;11:6601-6.

6. Lou G, Gao Y, Ning XM, Zhang QF. Expression and correlation of CD44v6, vascular endothelial growth factor, matrix metalloproteinase-2, and matrix metalloproteinase-9 in Krukenberg tumor. World J Gastroenterol. 2005;11:5032-6.

7. Han JC, Zhang KL, Chen XY, Jiang HF, Kong QY, Sun Y, et al. Expression of seven gastric cancer-associated genes and its relevance for Wnt, NF-kappaB and Stat3 signaling. APMIS. 2007;115:1331-43.

8. Zhou DH, Ma ZM, Chen Y. [Expression and clinical significance of CD44v6 and sCD44v6 in gastric carcinoma]. Zhonghua Zhong Liu Za Zhi. 2007;29:833-7.

9. Liang YZ, Fang TY, Xu HG, Zhuo ZQ. Expression of CD44v6 and Livin in gastric cancer tissue. Chin Med J (Engl). 2012;125:3161-5.

10. Ru Y, Zhang L, Chen Q, Gao SG, Wang GP, Qu ZF, et al. Detection and clinical significance of lymph node micrometastasis in gastric cardia adenocarcinoma. J Int Med Res. 2012;40:293-9.

11. Kurozumi K, Nishida T, Nakao K, Nakahara M, Tsujimoto M. Expression of CD44 variant 6 and lymphatic invasion: importance to lymph node metastasis in gastric cancer. World J Surg. 1998;22:853-7; discussion 7-8.

12. Saito H, Tsujitani S, Katano K, Ikeguchi M, Maeta M, Kaibara N. Serum concentration of CD44 variant 6 and its relation to prognosis in patients with gastric carcinoma. Cancer. 1998;83:1094-101.

13. Yamaguchi A, Goi T, Yu J, Hirono Y, Ishida M, Iida A, et al. Expression of CD44v6 in advanced gastric cancer and its relationship to hematogenous metastasis and long-term prognosis. J Surg Oncol. 2002;79:230-5.

14. Okayama H, Kumamoto K, Saitou K, Hayase S, Kofunato Y, Sato Y, et al. CD44v6, MMP-7 and nuclear Cdx2 are significant biomarkers for prediction of lymph node metastasis in primary gastric cancer. Oncol Rep. 2009;22:745-55.

15. Joo M, Lee HK, Kang YK. Expression of E-cadherin, beta-catenin, CD44s and CD44v6 in gastric adenocarcinoma: relationship with lymph node metastasis. Anticancer Res. 2003;23:1581-8.

16. Eom DW, Kang GH, Han SH, Cheon GJ, Han KH, Oh HS, et al. Gastric micropapillary carcinoma: A distinct subtype with a significantly worse prognosis in TNM stages I and II. Am J Surg Pathol. 2011;35:84-91.
