# Supplementary material for: Clinical and Prognostic Significance of HIF-1α, PTEN, CD44v6, and Survivin for Gastric Cancer: A Meta-Analysis
Source: PLoS One. 2014 Mar 19;9(3):e91842. doi: 10.1371/journal.pone.0091842 (PMC3960154; doi:10.1371/journal.pone.0091842)
Supplement: Flow Diagram S1 — PRISMA 2009 Flow Diagram for the Meta-Analyses. (DOC) [file pone.0091842.s004.doc]

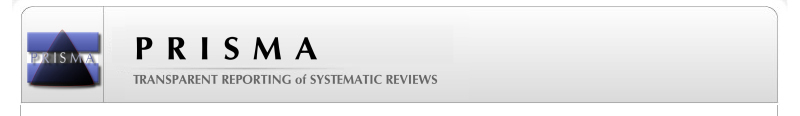
**PRISMA 2009 Flow Diagram**

**Screening**

**Included**

**Eligibility**

**Identification**

Records identified through database searching
(n = 864 )

Additional records identified through other sources
(n = 0 )

Records after duplicates removed
(n = 679 )

Records screened
(n = 421 )

Records excluded
(n =258 )

Full-text articles assessed for eligibility
(n = 208 )

Full-text articles excluded, with reasons
(n = 213 )

Studies included in qualitative synthesis
(n =89 )

Studies included in quantitative synthesis (meta-analysis)
(n = 73 )
